# Sleep structure of short-term insomnia disorder with mild cognitive impairment in older adults and their correlation with cognitive function: a case-control study

**DOI:** 10.3389/fnagi.2024.1507285

**Published:** 2025-01-03

**Authors:** Jinkun Zeng, Jia Wei, Ruobing Qi

**Affiliations:** Affiliated Mental Health Center & Hangzhou Seventh People’s Hospital, Zhejiang University School of Medicine, Hangzhou, China

**Keywords:** short-term insomnia disorder, mild cognitive impairment, polysomnography, older adults, case-control study

## Abstract

**Objectives:**

This study seeks to delineate the sleep architecture characteristics in older adults with short-term insomnia and mild cognitive impairment (MCI) and to explore their association with cognitive performance.

**Methods:**

Ninety elderly individuals with short-term insomnia were enrolled and stratified into two cohorts based on their Montreal Cognitive Assessment (MoCA) scores: the Short-Term Insomnia Group (STID) comprising 35 participants and the Short-Term Insomnia with Cognitive Impairment Group (STID-MCI) with 55 participants. Demographic data, Pittsburgh Sleep Quality Index (PSQI), MoCA, Hamilton Depression Rating Scale (HAMD-17), Hamilton Anxiety Rating Scale (HAMA), and polysomnography (PSG) parameters were compared between groups. Correlations between MoCA scores and PSG metrics were also analyzed.

**Results:**

No significant disparities were noted between groups in terms of HAMD-17, HAMA, and PSQI scores (*p* > 0.05). However, marked differences were identified in MoCA scores and its subdomains (*p* < 0.05). Significant variations were also observed in the duration and proportion of slow-wave sleep (N3) between groups (*p* < 0.05). In STID-MCI patients, memory scores correlated positively with N3 duration and percentage (*p* < 0.05), while verbal functions and attention were positively associated with rapid eye movement (REM) sleep duration.

**Conclusion:**

This study highlights the potential of PSG in the clinical assessment of cognitive function and underscores the need for targeted interventions to improve sleep quality in this vulnerable population.

## Introduction

According to the “Guidelines for the Diagnosis and Treatment of Insomnia Disorder in China (2017)” (Shuai and Bin, 2017), insomnia is classified into short-term, chronic, and other insomnia related disorders. Short-term insomnia disorder (STID), also known as acute insomnia, refers to insomnia that does not meet the frequency and duration criteria for chronic insomnia disorder (CID) but is associated with significant daytime functional impairment and clinical concern (Momin and Ketvertis, 2024). STID is typically related to psychological and environmental stressors, conflict, or significant emotional fluctuations (Riemann et al., 2015; Sutton, 2021). The one-year prevalence rate in adults is between 15 and 20% (Riemann et al., 2015), and, similar to CID, STID is more common among women and older adult (Qaseem et al., 2016).

Over the past decade, the association between insomnia and cognitive deficits has garnered increasing attention. Research indicates that patients with insomnia have a 1.5-fold higher risk of developing all-cause dementia compared to individuals without insomnia (Irwin and Vitiello, 2019; Zhang et al., 2021). Numerous studies suggest that patients with insomnia exhibit varying degrees of cognitive impairment with worse episodic memory function and attention compared to those without insomnia (Brownlow et al., 2020; Dopheide, 2020; Olaithe et al., 2018). Sleep is not currently mentioned as a modifiable risk factor for dementia (Livingston et al., 2020), and more research is needed to understand the contribution of sleep to cognitive deficits. Otherwise, in clinical practice, we observed that patients with short-term insomnia disorder who exhibited certain characteristic sleep structure abnormalities were more likely to report memory complaints. However, to date, no research has specifically addressed cognitive impairment associated with short-term insomnia.

According to the World Alzheimer Report 2023 (International, A. s. D, 2023), 55 million people worldwide were living with dementia in 2019, and this number is projected to grow exponentially due to the rapidly aging population. Since 2016, the conceptualization of AD has shifted toward a biological framework, particularly through the ATN model (Jack et al., 2024; Jack et al., 2016), which emphasizes biomarkers for amyloid (A), tau (T), and neurodegeneration (N) as central to defining the disease process. Despite significant progress in the diagnosis of Alzheimer’s disease, treatment still faces major challenges. Although certain monoclonal antibodies (such as Lecanemab and Donanemab) have shown some efficacy in reducing amyloid plaque deposition, the overall improvement in cognitive function remains limited. The cost of monoclonal antibody treatments is high, which may lead to an economic burden on patients and the healthcare system. Additionally, current research indicates that monoclonal antibodies are most effective in patients with early-stage Alzheimer’s disease, so they may not achieve the desired results in moderate to advanced patients (Espay et al., 2024; Terao and Kodama, 2024). In light of the treatment challenges of Alzheimer’s disease, clinical interventions and drug developments have shifted focus to earlier stages, such as MCI and even subjective cognitive decline (SCD) (Anderson, 2020; Graff-Radford et al., 2021; Wang et al., 2020). Therefore, successful implementation of early detection and prevention strategies at the MCI stage is of great importance.

Previous studies using polysomnography (PSG) in the general population have demonstrated differences in sleep characteristics (Vaughn and Giallanza, 2008), such as reduced rapid eye movement (REM) sleep, increased fragmentation of slow-wave sleep, or more severe sleep-disordered breathing indices between participants with MCI and those with intact cognition (Shu et al., 2022). A study examining sleep architecture differences between insomnia patients with MCI and those with intact cognition found that, in patients with insomnia disorder, sleep duration, sleep fragmentation, sleep efficiency, N1%, and N3% were independently associated with the presence of a MCI (Zhang et al., 2021). However, no research has yet examined the sleep structures of short-term insomnia patients with mild cognitive impairment.

Therefore, we hypothesize that short-term insomnia individuals accompanied by cognitive impairments have distinct sleep architecture features, which may be the primary reason for their cognitive deficits. In this study, we aimed to compare objective sleep features measured by PSG in short-term insomnia disorder patients with MCI to those with normal cognition, and to analyze the correlation between cognitive impairment and sleep characteristics in STID-MCI. Clarifying the association between sleep features and MCI in patients with short-term insomnia disorder could promote clinicians to more closely examine their patients’ cognitive function.

## Methods

### Sample size calculation

In our study, the sample sizes of the STID and STID-MCI groups were calculated using PASS v21.0.3. According to the study by Maestri et al. (2015), the average and standard deviation of Sleep stage N3 (%) for the intact cognition (HE) group and mild cognitive impairment (MCI) group are 23.5 ± 4.8 and 19.5 ± 4.3, respectively. Therefore, we substituted the average and standard deviation of Sleep stage N3 (%) reported in the study by Maestri et al. (2015), into PASS V21.0.3 to calculate the validity. Group sample sizes of 33 and 33 achieve 90.532% power to reject the null hypothesis of equal means when the population mean difference is μ1–μ2 = 19.5–23.5 = −4 with a standard deviation for both groups of 4.89 and with a significance level (alpha) of 0.05 using a two-sided two-sample equal-variance *t*-test. In this study, we finally included 35 STID and 55 SITD-MCI participants in our study.

### Participants

This study was conducted in the Department of Geriatric Psychiatry of Hangzhou Seventh People’s Hospital and was approved by the Medical Ethics Committee of Hangzhou Seventh People’s Hospital (No. 2021-025). Written informed consent was obtained from all participants.

This study enrolled 90 elderly patients diagnosed with short-term insomnia disorder (STID) between September 2021 and January 2024. Inclusion criteria were: (1) age ≥ 60 years; (2) sleep disturbances lasting less than 3 months; (3) Pittsburgh Sleep Quality Index (PSQI) score ≥ 7; (4) Hamilton Depression Rating Scale (HAMD-17) score < 16; (5) Hamilton Anxiety Rating Scale (HAMA-14) score < 14; (6) absence of other sleep disorders, such as sleep apnea or restless legs syndrome. Participants were excluded if they met any of the following criteria: (1) presence of severe physical illnesses that could adversely affect cognitive function; (2) concurrent psychiatric disorders or other sleep disorders that might contribute to insomnia. MCI was diagnosed if the subject met the following criteria: memory complaint, normal activities of daily living, normal general cognitive function, abnormal memory for age, and a lack of dementia (Petersen et al., 1995). The Patients with Montreal Cognitive Assessment (MoCA) scores of 18–25 were assigned to the STID combined MCI group (*n* = 55), and those with MoCA scores of 26–30 were assigned to the STID group with normal cognition (*n* = 35).

### Demographic data

Demographic information was collected through questionnaires and included gender, age, BMI, years of education, smoking history (defined as smoking more than one cigarette per day for at least 6 months), and alcohol consumption (defined as drinking more than once in the past month).

### Neuropsychological assessment

In the morning before PSG recording, patients underwent some neuropsychological assessments. The evaluation included the 17-item Hamilton Rating Scale for Depression (HAMD-17) and the 14-item Hamilton Anxiety Rating Scale (HAMA-14) to assess depressive and anxiety symptoms. Cognitive function was evaluated using the MoCA (Changsha edition).

### Sleep evaluation

Overnight PSG monitoring was conducted for each participant between 4:00 PM and 5:00 PM in the neurology department, administered by trained medical technicians using standard techniques. PSG recordings included continuous monitoring from six electroencephalographic leads (international 10–20 system: F3-A2, F4-C1, C3-A2, C4-A1, O1-A2, and O2-A1), two electro-oculographic leads (ROC-A1, LOC-A2), thermistors for nasal and oral airflow, strain gauges for thoracic and abdominal excursion and finger pulse oximetry.

Sleep indices recorded included sleep architecture data – such as time in bed, total sleep time (TST), sleep latency, sleep efficiency (SE), time in each stage, arousal events (e.g., arousal index), cardiac events (e.g., average heart rate during sleep), and respiratory events (e.g., AHI). Scoring was performed according to Version 2.4 of the AASM Manual for the Scoring of Sleep and Associated Events: Rules, Terminology and Technical Specifications (Berry et al., 2017). Sleep quality was further evaluated using the Pittsburgh Sleep Quality Index (PSQI) in the morning following PSG monitoring.

### Statistical analysis

Data were analyzed using SPSS 25.0 statistical software. Normally distributed metric data are presented as mean ± standard deviation, while non-normally distributed metric data are presented as median (interquartile range). Categorical data are expressed as percentages (%). Comparisons between two groups for normally distributed variables were performed using the *t*-test, and for non-normally distributed variables between two groups the rank-sum test was used.

## Results

### Comparison of demographic profiles

A total of 90 eligible short-term insomnia disorder patients were included in the study: 35 patients with STID and 55 participants with STID-MCI. The STID-MCI and STID participants exhibited similar demographic characteristics (Table 1). There were no differences between the groups in age, sex, BMI, education level, smoking status, alcohol intake, or depression and anxiety symptoms.

**Table 1:** Demographic and clinical characteristics in the short-term insomnia disorder (STID) and the short-term insomnia disorder with mild cognitive impairment (STID-MCI) group.

Variable	Mean (SD), *n* (%) or median (interquartile range)		*Χ* ^2^ */t*	*P*
	STID (*n* = 35)	STID-MCI (*n* = 55)		
Female, *n* (%)	21(60%)	31(56.4%)	0.116	0.733^#^
Age, years	64.17 ± 4.69	66.07 ± 6.07	−1.576	0.119
Education, years	6(3.5,9)	6(3,9)	0.823	0.413
BMI, kg/m^2^	22.88 ± 3.11	22.61 ± 6.07	0.412	0.681
Smoking, *n* (%)	2(5.7%)	5(9.1%)	0.340	0.56^#^
Drinking, *n* (%)	1(2.9%)	5(9.1%)	1.336	0.248^#^
HAMD-17 score	7.94 ± 3.36	8.07 ± 3.33	−0.180	0.858
HAMA-14 score	6.83 ± 3.23	7.02 ± 2.79	−0.295	0.769

All test statistics are Independent *t*-tests unless otherwise specified. Values are the mean (SD), *n* (%) or median (interquartile range). BMI, body mass index; HAMD-17, 17-item Hamilton Rating Scale for Depression; HAMA-14, 14-item Hamilton Anxiety Rating Scale; SD, standard deviation, ^#^Chi-squared analyses were used.

Compared to the STID patients, patients in the STID-MCI group had significantly lower scores on the Montreal Cognitive Assessment (MoCA) subdomains, including spatial/executive function, memory, attention, language fluency, and abstract thinking (Table 2).

**Table 2:** Comparisons of the Montreal Cognitive Assessment (MoCA) and the Pittsburgh sleep quality index (PSQI) between the STID group and STID-MCI group.

Scales	STID (*n* = 35)	STID-MCI (*n* = 55)	*Z*	*p*
MoCA score	27.06 ± 0.99	22.04 ± 2.27	14.356	<0.001
Spatial/executive ability	4.06 ± 0.93	2.35 ± 1.23	7.444	<0.001
Naming	2.97 ± 0.16	2.87 ± 0.43	1.519	0.133
Memory	3.46 ± 0.91	2.18 ± 1.34	5.334	<0.001
Attention	5.60 ± 0.48	4.78 ± 1.18	4.572	<0.001
Language fluency	2.29 ± 0.51	1.85 ± 0.55	3.731	<0.001
Abstract thinking	1.86 ± 0.35	1.36 ± 0.80	3.991	<0.001
Orientation	5.97 ± 0.16	5.91 ± 0.34	1.134	0.260
PSQI	16.09 ± 2.13	15.38 ± 3.78	1.003	0.319
Subjective sleep quality	2.57 ± 0.77	2.46 ± 0.77	0.647	0.520
Sleep quality	2.63 ± 0.59	2.37 ± 0.85	1.677	0.097
Sleep duration	2.71 ± 0.57	2.48 ± 0.69	1.722	0.089
Habitual sleep efficiency	2.74 ± 0.56	2.70 ± 0.63	0.298	0.767
Sleep disturbances	1.46 ± 0.56	1.39 ± 0.59	0.540	0.591
Use of sleep medicine	2.29 ± 1.07	2.30 ± 1.16	−0.043	0.966
Daytime dysfunction	1.69 ± 0.96	1.96 ± 1.06	−1.246	0.216

### Sleep characteristics

There were no significant differences between the STID-MCI and STID groups in self-reported sleep quality, either in the total score or individual items of the Pittsburgh Sleep Quality Index (PSQI) (Table 2). Objective sleep indices obtained from polysomnography (PSG) are presented in Table 3. Compared to the STID group, the STID-MCI group demonstrated reduced total sleep time (TST), lower sleep efficiency (SE), decreased rapid eye movement (REM) sleep duration, and a lower percentage of N3 sleep (slow-wave sleep). Specifically, TST was shorter in the STID-MCI group than in the STID group (334.5 min vs. 361.5 min vs. 435.7 min), and SE was lower in the STID-MCI group compared to the STID group (75% vs. 79.8%). REM sleep duration was decreased in the STID-MCI group compared to the STID group (56.5 min vs. 69.5 min). Additionally, the percentage of N3 sleep was lower in the STID-MCI group than in the STID group.

**Table 3:** Sleep parameters in STID and STID-MCI groups.

Scales	STID (*n* = 35)	STID-MCI (*n* = 55)	*Z*	*p*
TST, min	435.75(404,453.75)	361.5(320,439)	−3.319	0.001
TWT, min	108.75(61,135.25)	113(77.75,197.5)	1.853	0.064
WASO, min	80.5(47.75,109.75)	92(58.5,164.25)	1.782	0.075
Sleep efficiency (SE) (%)	79.78(75.70,88.11)	75(63.11,84.08)	−2.065	0.039
N1 duration	51.5(33.75,71)	59.5(39.75,70.75)	−0.062	0.950
N2 duration	299.5(224.25,316.25)	235(202.25,302.5)	−1.813	0.070
N3 duration	0(0,32)	0(0,1.75)	2.338	0.022
REM duration	69.51(51,83.25)	56.5(32,75)	−2.185	0.032
N1, % of TST	12.4(7.9,16.85)	15.4(10.25,19.55)	1.192	0.233
N2, % of TST	66.7(54.7,73.75)	67.6(59.65,74.68)	0.521	0.602
N3, % of TST	0(0,6.55)	0(0,0.6)	2.224	0.029
REM, % of TST	16.75(11.7,19.65)	16.2(8.7,21.8)	−1.047	0.295
SOL, min	17.25(4.25,28.5)	19(8.75,31.75)	0.922	0.357
AHI, events/h	2.58(0.40,5.07)	1.65(0.41,5.21)	−0.190	0.849

Data are expressed as the median (interquartile range). All test statistics are Mann–Whitney *U* test. AHI, apnea-hypopnea index; N1, stage 1 of non–rapid eye movement; N2, stage 2 of non–rapid eye movement; N3, stage 3 of non–rapid eye movement sleep; REM, rapid eye movement sleep; SE, sleep efficiency.

### Analysis of the association between objective sleep characteristics and cognitive function in short-term insomnia disorder patients with MCI

In the STID-MCI group, Spearman correlation analyses were conducted to assess the relationships between PSG parameters and MoCA total and subdomain scores. The memory subdomain score was positively correlated with N3 sleep duration (*r* = 0.291, *p* = 0.031) and the percentage of N3 sleep (*r* = 0.301, *p* = 0.026). Additionally, the orientation subdomain score was positively correlated with the percentage of REM sleep (*r* = 0.276, *p* = 0.042) (Table 4). No significant correlations were found between other PSG parameters and cognitive function scores.

**Table 4:** Correlation analysis of PSG parameters and MoCA total scores and it’s items in the STID-MCI group.

PSG parameters	MoCA total scores	Spatial/executive ability	Naming	Memory	Attention	Language fluency	Abstract thinking	Orientation
TST	0.043	−0.045	−0.122	−0.040	0.124	0.144	0.100	0.092
TWT	−0.151	0.106	−0.071	−0.189	−0.064	−0.126	−0.101	−0.222
WASO	−0.193	0.031	−0.133	−0.158	−0.102	−0.123	−0.098	−0.182
SOL	0.058	0.194	0.121	−0.117	0.070	−0.038	−0.032	−0.143
SE	0.002	−0.065	−0.053	−0.083	0.009	0.015	−0.037	0.222
N1 duration	0.019	0.066	0.062	−0.067	−0.154	0.025	−0.009	0.067
N2 duration	−0.034	−0.130	−0.123	0.057	0.094	0.055	0.123	−0.054
N3 duration	−0.060	−0.101	−0.034	0.291*	0.031	−0.129	−0.084	−0.088
REM duration	0.164	0.145	−0.055	0.257	0.238	0.207	−0.002	0.254
N1%	0.039	0.068	0.101	−0.008	−0.177	0.030	−0.063	0.055
N2%	−0.138	−0.170	−0.061	0.095	0.013	−0.098	0.090	−0.194
N3%	−0.048	−0.107	−0.061	0.301*	0.013	−0.098	0.090	−0.194
REM%	0.187	0.255	−0.040	0.026	0.233	0.179	−0.019	0.276*
AHI	−0.030	0.040	0.022	0.054	−0.120	−0.105	0.007	0.021

* *p* < 0.05 (two-tailed), the correlation is significant.

## Discussion

In this study, we examined the objective and self-reported sleep characteristics of short-term insomnia disorder participants with MCI and those with intact cognition. There were no significant differences in self-reported sleep quality between the STID-MCI and STID groups. However, PSG revealed that TST, SE, N3 duration, REM duration and N3% were independently associated with the presence of MCI in the short-term insomnia disorder population. Lower N3% and decreased N3 duration were risk factors for memory impairment, and decreased REM% was associated with impaired orientation ability in STID-MCI patients. The discrepancy between self-reported and PSG findings may result from sleep misperception in insomnia or cognitive deficits in MCI, indicating that PSG monitoring may be sufficiently sensitive to reveal characteristic sleep abnormalities in patients with MCI (Zhang et al., 2021).

Most epidemiological studies on MCI have not specifically targeted short-term insomnia populations (Hamdy et al., 2018; Pak et al., 2020). However, consistent with our findings, previous studies have indicated that MCI patients experience less slow-wave sleep (SWS) (Carnicelli et al., 2019; Haba-Rubio et al., 2017; Hita-Yañez et al., 2013), poorer sleep continuity (De Guia et al., 2024; Uddin et al., 2020) and shorter total sleep time compared to healthy individuals (Tang et al., 2024). A large prospective study measuring sleep patterns through actigraphy reported that increased sleep fragmentation is associated with a higher risk of Alzheimer’s disease (AD) and a faster decline in cognitive abilities among older adults (Agudelo et al., 2021). A recent meta-analysis of objective measurements in patients with MCI showed significant changes in sleep structure compared to healthy controls, including reduced TST, lower SE, prolonged sleep onset latency (SL), prolonged REM latency, reduced REM sleep, increased N1 sleep, and more severe hypoxemia (Cai et al., 2020). A recent meta-analysis aimed at clarifying the differences in sleep structure between MCI patients and cognitively normal elderly individuals found that MCI patients had significantly lower SE – a key indicator of sleep quality – and significantly reduced SWS (D'Rozario et al., 2020). However, there were no significant differences in TST, SL, AIH, SWS%, REM%, and WASO between the two groups. These findings are consistent with our results in STID-MCI and STID patients. Our findings suggest that STID patients with MCI exhibit distinct sleep structure patterns compared to those without cognitive impairment. Clinically, it is important to consider cognitive assessment when addressing short-term insomnia patients with similar sleep structures.

Mounting epidemiologic evidence implicates disturbed sleep or lack of sleep as risk factors for AD (Bubu et al., 2017; Holingue et al., 2018). A case–control study indicated that insufficiency of SWS may underlie the association between insomnia and deficits in executive attention control. Memory impairment in aging has also been linked to suppressed slow waves, consistent with our findings that memory function is positively correlated with N3 sleep and N3%. A possible explanation for the relationship between insomnia and cognitive decline observed in our study is that accumulation of amyloid-*β* and tau aggregates—two histopathological markers of AD—is correlated with decreased SWS (Lee et al., 2021). Moreover, recent studies suggest that loss of slow-wave sleep could be associated with decreased cerebrospinal fluid flow, leading to reduced clearance of protein aggregates, which is a risk factor for cognitive impairment (Fultz et al., 2019; Olsson et al., 2018). High quality of sleep may mitigate the impact of pathophysiological mechanisms in mild cognitive impairment (MCI) through functional connectivity reorganization of neural networks underlying higher cognitive functions. A study of 38 patients with MCI stratified into high and low quality of sleep in accordance with a self-reported questionnaire for sleep habits, and 38 controls underwent resting-state functional magnetic resonance imaging found that high quality of sleep was associated with increased frontoparietal network (FPN) connectivity among patients with MCI. Moreover, a positive coupling of connectivity between networks was found in MCI reporting high quality of sleep, congruently with the pattern observed in controls, whereas this coupling was disrupted in MCI with low quality of sleep (Pini et al., 2020). This study explains the relationship between sleep and mild cognitive impairment from a neuroimaging perspective.

This study found significant differences in N3 and REM sleep between STID-MCI and STID patients, suggesting that changes in sleep architecture may contribute to cognitive decline. In a longitudinal study involving 2,238 healthy older adults and 655 MCI patients over 4 years, increased sleep latency and low sleep efficiency were identified as early clinical indicators of cognitive decline, consistent with our results (Suh et al., 2018). Sleep deprivation or restriction can activate a systemic inflammatory response and increase pro-inflammatory cytokines, leading to dysfunctional microglia, neuronal damage, and reduced clearance of *β*-amyloid due to local inflammation in the central nervous system, thereby impacting cognitive function (Sang et al., 2023; Targa et al., 2021).

The main strengths of this study include: (1) all patients with STID were diagnosed by physicians strictly according to clinical standards, based not only on insomnia complaints but also on the effects on their daily lives; (2) PSG monitoring was conducted to provide a detailed records of sleep parameters. However, some limitations should be considered: First, this study was not prospective, so we cannot conclude whether sleep disturbances preceded or followed the development of MCI. Second, we could not completely rule out the possibility of residual confounding by unmeasured factors. Our study is merely a simple correlational study, and further research is needed to clarify the relationship between different sleep stages and cognition. In conclusion, lower sleep efficiency, short sleep duration, and reduced SWS may be related to cognitive impairment in patients with short-insomnia disorder. Although these findings may not be exclusive to short-term insomnia patients with MCI, they highlight the importance of clinicians paying more attention to the cognitive status of patients with short-term insomnia.

## Data Availability

The original contributions presented in the study are included in the article/supplementary material, further inquiries can be directed to the corresponding author.
